# Alcohol Consumption and Development of Acute Respiratory Distress Syndrome: A Population-Based Study

**DOI:** 10.3390/ijerph6092426

**Published:** 2009-09-10

**Authors:** Lokendra Thakur, Marija Kojicic, Sweta J. Thakur, Matthew S. Pieper, Rahul Kashyap, Cesar A. Trillo-Alvarez, Fernandez Javier, Rodrigo Cartin-Ceba, Ognjen Gajic

**Affiliations:** Division of Pulmonary and Critical Care Medicine, College of Medicine, Mayo Clinic, Rochester, MN; (M.E.T.R.I.C.) Multidisciplinary Epidemiology and Translational Research in Intensive Care, 200 1st St SW, Rochester, MN 55905, USA; E-Mails:lokendrat2001@yahoo.com (L.T.);kojicic.marija@gmail.com (M.K.);thakur.sweta@mayo.edu (S.J.T.);pieper.matthew@mayo.edu (M.S.P.);kashyap.rahul@mayo.edu (R.K.);trillomd@stanford.edu (C.A.T.);jfdez@clinic.ub.es (F.J.);cartinceba.rodrigo@mayo.edu (R.C.)

**Keywords:** alcohol, ARDS, population

## Abstract

This retrospective population-based study evaluated the effects of alcohol consumption on the development of acute respiratory distress syndrome (ARDS). Alcohol consumption was quantified based on patient and/or family provided information at the time of hospital admission. ARDS was defined according to American-European consensus conference (AECC). From 1,422 critically ill Olmsted county residents, 1,357 had information about alcohol use in their medical records, 77 (6%) of whom developed ARDS. A history of significant alcohol consumption (more than two drinks per day) was reported in 97 (7%) of patients. When adjusted for underlying ARDS risk factors (aspiration, chemotherapy, high-risk surgery, pancreatitis, sepsis, shock), smoking, cirrhosis and gender, history of significant alcohol consumption was associated with increased risk of ARDS development (odds ratio 2.9, 95% CI 1.3–6.2). This population-based study confirmed that excessive alcohol consumption is associated with higher risk of ARDS.

## 1. Introduction

Alcohol is one of the most commonly used drugs worldwide. Lifetime prevalence of alcohol use is 66% in the United States, with nearly 50% of adult Americans using alcohol sometime during the previous 12 months [1]. Drinking more than two glasses of alcohol per day (>14 drink a week) has been shown to be associated with increased mortality [2,3]. Epidemiological studies estimate that 13% of Americans will meet the diagnostic criteria for alcohol dependence during their lifetime. The worldwide prevalence of alcohol abuse and dependence in hospitalized patients is similar [4,5]. More than 20 % of inpatients on medical and surgical services in United States have positive alcohol abuse history. Up to 42% of general hospital inpatients are reported to have alcohol-related disorders [4,6]. The hospital mortality of patients with prior history of alcohol abuse is higher as compared to non-alcoholics [7].

ARDS is a severe form of acute lung injury that usually develops soon after major injury or illness [8–12]. The reported incidence of ARDS ranges from 8.3 to 58.7 per 100,000 person-years [13,14]. Despite recent advances in our understanding of the pathophysiology and treatment of ARDS, mortality has remained very high [12]. In ARDS, lung tissue suffers injury by diffuse activation of the inflammatory pathways resulting in hypoxemia. Networks of inflammatory cells and cytokines contribute to diffuse damage to the lung parenchyma, leakage of fluid into the alveolar space, thrombosis of small vessels and inflammatory cellular infiltration.

Prolonged and heavy amount of alcohol consumption has a detrimental effect on multiple organ systems and has been described as an important risk factor for the development of ARDS [15–17]. Previous studies that implicated alcohol consumption as a risk factor for ARDS did not include a population-based approach. Hence, we decided to perform a community cohort study to measure the impact of alcohol abuse on the development of ARDS in critically ill patients.

## 2. Methods

A retrospective observational cohort study of critically ill Olmsted County residents (age ≥18 years) who were admitted to medical and surgical intensive care units (ICU) at the two Mayo Clinic hospitals in Rochester (Minnesota, USA) during 2006 was undertaken. Patients with possible ARDS were first identified with the help of an electronic alert system. This system has been validated in previous publications against the gold standard of prospective assessment by trained intensivist researchers, blinded to the electronic alert (negative predictive value of the electronic alert 99%; 95% CI, 98–100%) [18]. Electronic medical records of identified patients were reviewed by trained investigators in order to confirm the diagnosis of ARDS. The inter-observer agreement in applying the ARDS definition was calculated for the purpose of a previous study (Kappa value of 0. 83).

Alcohol consumption was quantified based on patient and/or family provided information at the time of hospital admission. Use of surrogates to answer standardized alcohol questionnaires has been previously validated and correlates well with the primary respondents [19,20]. The Institutional Review Board approved the study protocol and waived the need for informed consent. Patients who denied prior authorization for the use of their medical records for research, as required by Minnesota law, and those who did not provide information about alcohol consumption were excluded.

## 3. Definitions

Acute Respiratory Distress Syndrome was defined according to standard consensus conference definition as presence of: PaO2/FiO2% < 200 mmHg, bilateral lung infiltrates on chest radiograph, pulmonary artery wedge pressure <18 mmHg or no clinical evidence of left atrial hypertension [21,22].

Excessive alcohol consumption (alcohol abuse) was defined as a known diagnosis of chronic alcoholism, a previous admission for alcohol detoxification or alcohol withdrawal [23], or a reported alcohol consumption of more than two drinks per day or >14 drinks/week [2,3,24,25]. The Mayo Clinic hospitals use a standardized patient provided information (PPI) form that is filled out by the patient or a surrogate at the time of each hospital visit. The form contains not only the qualitative but also quantitative information (number of drinks a day). In a case of multiple admissions and discrepant results, the highest amount of alcohol consumption was recorded. Smoking was defined as current smoker or history of more than 20 pack-years of smoking. Standard clinical definitions were used to define sepsis [26], shock [27], pneumonia [28], trauma [29], pancreatitis [30] and high-risk surgery [31,32]. All data were collected from existing electronic medical records. The data included information before and at the time of hospital admission, and daily after admission until the outcome is reached.

## 4. Statistical Analysis

All data are presented as mean (standard deviation) (SD), median (interquartile range) (IQR), counts or percentages. We used unpaired Student’s t test to compare continuous variables with normal distribution and Mann-Whitney U test for skewed distribution. For comparison of categorical variables, we used chi-square test if the number of elements in each cell is 5 or higher and Fisher’s exact test otherwise. To determine the independent impact of alcohol consumption on the ARDS incidence, we created a multiple logistic regression model by entering the co-morbid risk factors as co-variables, taking into consideration colinearity and clinical plausibility. A p-value of <0.05 was considered statistically significant. JMP statistical software (SAS Corporation, Cary, NC, USA) was used for all data analysis.

## 5. Results

**Figure 1.**
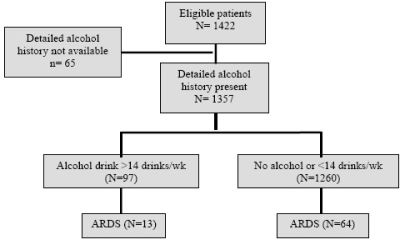
Study outline.

A total of 1,422 Olmsted County patients admitted to the medical and surgical ICUs in 2006 were enrolled in the study. For 65 patients a detailed alcohol history was not available, so they were excluded from the analysis. A history of significant alcohol consumption was reported in 97 patients (7%) (Figure 1).

Table 1 describes baseline characteristics and outcome of patients who did or did not report excessive alcohol consumption. Excessive alcohol consumption was associated with male gender, smoking, aspiration and liver cirrhosis.

**Figure 2.**
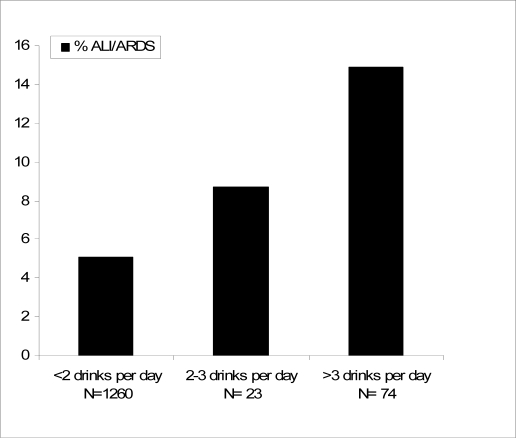
Dose response relationship of alcohol consumption on the development of ARDS.

During hospital stay, ARDS developed in 13 out of 97 (13%) patients with a significant alcohol consumption history, compared to only 64 out of 1,260 (5%) non alcoholic or patients consuming <14 drink/week (p < 0.001). When stratified according to amount of alcoholic drinks per day alcohol consumption and the development of ARDS demonstrated a dose response relationship (Figure 2). This was held true when adjusted for other known ARDS risk factors (aspiration, chemotherapy, high-risk surgery, pancreatitis, sepsis, shock) (p = 0.008). Patients who reported drinking less than two drinks per day (<14 drinks per week), had a similar incidence of ARDS compared to non drinkers (5.6 %).

Since the vast majority of patients who reported excessive alcohol consumption were also smokers we performed a stratified subset analysis of 350 patients who had a positive history of alcohol consumption and/or smoking. Alcohol was found to be more contributory to the development of ARDS than smoking.

To further investigate the role of potential confounding variables, a multivariate logistic regression model was fit for the development of ARDS. After adjusting for differences in well known underlying ARDS risk factors (aspiration, chemotherapy, high-risk surgery, pancreatitis, sepsis, shock), smoking, cirrhosis and gender, the effects of a positive history of significant alcohol consumption on the development of ARDS remained significant (Odds ratio 2.9, 95% CI 1.3–6.2) (Table 2).

## 6. Discussion

In this retrospective population based study we observed a positive association between the history of significant alcohol consumption and the development of ARDS in critically ill patients. The association of alcohol and ARDS development demonstrated a dose response relationship and was independent in multivariate analysis after adjusting for co-exposures and other known causes of ARDS.

These results confirm previous reports that showed strong association of alcohol and acute lung injury and ARDS [23,33] but none of the existing studies used a population based approach. The previous studies were done in tertiary care centers that receive more sick patients from different demography and are prone to referral bias, so the results are difficult to generalize in a community.

In patients with history of chronic alcohol abuse, lungs are more vulnerable to oxidative stress and injury most likely due to lack of Glutathione-GSH to scavenge the oxygen free radicals. Decreased levels of the free radical scavengers increase the inflammatory injury to the lung and may explain the detrimental effects of alcohol consumption. There is evidence that chronic alcohol consumption decreases the level of glutathione promoting inflammation and remodeling of the lung tissue [16,34].

Patients with heavy alcohol consumption are exposed to multiple confounding factors. Some factors like aspiration, liver cirrhosis, coagulopathy, multiple transfusions and hypoalbuminemia, as well as different type and severity of alcohol abuse have not been investigated thoroughly. All these factors may be contributing to the development of ARDS in prolonged or heavy alcohol consumers. The individual contribution of these factors on the development of ARDS requires prospective study.

One of the limitations in comparing the results from different studies linking alcohol and ARDS is that the measurement of significant alcohol consumption is not standardized. Questionnaires like the CAGE and SMAST scoring systems are difficult to administer in critically ill patients, and the investigators are forced to rely on patient and the family provided information documented in the medical records. The definition used by Moss *et al*. [23] included patients admitted to the hospital with the diagnosis of chronic alcoholism, history of admission to an alcohol detoxification center or prior hospital admission for alcohol withdrawal. Using these criteria would exclude a proportion of the general population who uses alcohol significantly since it has been shown that only 10 percent of alcoholics are referred for alcohol related assessment and treatment [35]. For the purpose of this study, we used NIAAA (National Institute on Alcohol Abuse and Alcoholism) criteria, which are easy to follow and also encompass asymptomatic chronic alcohol users. Another report defined alcohol abuse as 60 g/day for several months [36]. According to NIAAA criteria it would come as four drinks per day (28 drinks/week) which is double our study criteria.

In our study, the prevalence of excessive alcohol use was lower than previously reported. This is not surprising as clinicians often underdiagnose alcoholism [37,38]. In addition, families have a tendency to underreport the volume of EtOH consumption. Indeed, studies have found that collateral informants are able to report the frequency of use accurately (i.e. daily or every other day or only on weekends) but tend to underreport the volume of use (two beers/day versus four beers/day [20,39,40]. The potential imprecision in volume of use to some extent diminishes the inferences from the dose-response relationship in Figure 2.

The univariate analysis failed to show a significant association between trauma and alcohol consumption. This could be due to a fact that previous studies were done at tertiary care centers located in urban areas where alcohol use disorders and major trauma are abundant.

The association between smoking and alcohol confounds the relationship between alcohol and ARDS development. Although our stratified analysis suggests that alcohol consumption is more contributory, the limited number of patients precluded meaningful analysis of the interaction between the two exposures.

Our study is limited by a relatively small number of patients and a single suburban population. In addition, the information on alcohol consumption collected retrospectively using hospital electronic medical records may have a limited precision. The data on severity of illness were not available. Observational nature of the study limits the causal inferences.

Our results are important for several reasons. Mortality of ARDS has remained high even despite the advancement of medical knowledge and technology, so it is essential to establish a preventive strategy in a population with high risk of ARDS development. In addition, physicians and patients should be aware of this additional risk of excessive alcohol consumption.

In summary, a history of chronic and significant alcohol use is associated with the development of ARDS in critically ill patients from suburban community. Prospective studies utilizing universal diagnostic criteria of significant alcohol consumption will need to be used for testing potential ARDS prevention strategies in this vulnerable population.

## Figures and Tables

**Figure 3.**
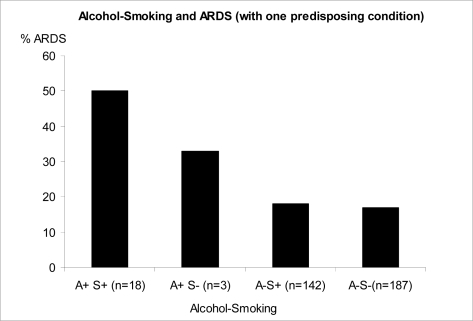
Frequency of ARDS according to alcohol and smoking history A-alcohol, S-smoking (p < 0.005 Chi Square test; no significant differences were found between A+S-and other subgroups).

**Table 1.** Differences in alcohol consumption and clinical characteristics of the patients.

	**Alcohol consumption >14 drinks/wk N = 97**	**No alcohol consumption or <14 drinks/wk N = 1,260**	**P value**

Age	55 (44 to 69)	66 (50 to 79)	<0.001
APACHE III scores (n = 632)	35 (26 to 61)	51 (36 to 68)	0.002
Male Gender, n (%)	82 (85)	624 (50)	<0.001*
Aspiration n (%)	7 (7)	32 (3)	<0.001*
Chemotherapy, n (%)	1 (1)	15 (1)	0.88
Cirrhosis, n (%)	9 (9)	8 (1)	<0.001*
Diabetes, n (%)	16 (16)	286 (23)	0.16
High Risk Surgery, n (%)	12 (12)	219 (17)	0.21
Pancreatitis, n (%)	1 (1)	4 (0)	0.26
Pneumonia, n (%)	10 (10)	99 (8)	0.39
Sepsis, n (%)	9 (9)	104 (8)	0.72
Shock, n (%)	7 (7)	86 (7)	0.88
Smoking, n (%)			<0.001*
Never	9 (9)	612 (49)	
Past	30 (31)	463 (37)	
Current	58 (60)	185 (14)	
Trauma, n (%)	4 (4)	50 (4)	0.93
Hospital Death, n (%)	7 (7)	114 (9)	0.54

**Table 2.** Factors associated with ARDS development.

**Variables**	**Univariate analysis**		**Multivariate analysis**	

	**Odds ratio**	**Confidence Interval (C.I.)**	**Odds ratio**	**Confidence Interval (C.I.)**

Alcohol Consumption >14 drinks a week	3.0	1.5–5.3	2.9	1.3–6.2
Aspiration	8.6	4–17.3	7.2	2.9–16.7
Chemotherapy	2.4	0.4–8.8	6.2	0.9–25.2
High Risk Surgery	3.2	1.9–5.2	4.8	2.7–8.3
Smoking (pack years)	3.2	0.7–11.9	1.0	0.99–1.01
Pancreatitis	4.2	0.2–28.8	2.9	0.1–27.8
Pneumonia	1.2	0.5–2.4	1.2	0.4–2.6
Sepsis	10.5	6.3–17.4	8.4	4.5–15.5
Shock	6.8	3.9–11.7	2.8	1.4–5.5
